# Purine Metabolism Pathway Influence on Running Capacity in Rats

**DOI:** 10.3390/metabo15040241

**Published:** 2025-04-02

**Authors:** Dengbo Chen, Christian Noble Biney, Qian Wang, Mingzheng Cai, Shi Cheng, Wentao Chen, Jinrui Zhang, Junran Zhao, Yuhan Zhang, Wenzhong Zhang

**Affiliations:** 1 School of Public Health and Preventive Medicine, Wannan Medical College, Wuhu 241002, China; 20239067@stu.wnmc.edu.cn (D.C.); 20248002@stu.wnmc.edu.cn (C.N.B.); 22109010121@stu.wnmc.edu.cn (M.C.); 20239066@stu.wnmc.edu.cn (S.C.); 20239089@stu.wnmc.edu.cn (W.C.); 21109010160@stu.wnmc.edu.cn (J.Z.); 22109010028@stu.wnmc.edu.cn (J.Z.); 22109020026@stu.wnmc.edu.cn (Y.Z.); 2 School of Public Health, Shandong Second Medical University, Jinan 252422, China; 20220909@stu.sdsmu.edu.cn

**Keywords:** SD rat model, metabolomics profiling, purine metabolism, inosine triphosphate (ITP), running capacity

## Abstract

**Background**: The natural differences in running capacities among rats remain poorly understood, and the mechanisms driving these differences need further investigation. **Methods**: Twenty male Sprague-Dawley (SD) rats were selected. High and low running capacity rats were identified using Treadmill Exhaustion Tests. Peripheral blood was collected for serum isolation, followed by a metabolomics analysis using LC-MS/MS. Data were preprocessed, and a principal component analysis (PCA) and a partial least squares-discriminant analysis (PLS-DA) were applied to identify metabolic profile differences. Significant metabolites were screened, and a pathway enrichment analysis was conducted using the KEGG database to determine key metabolic pathways. Forty SD rats (equal male and female) were randomly divided into an inosine triphosphate (ITP) group (24.29 mg/kg.bw daily) and a control group. Running capacity was assessed after one week of continuous treatment. **Results**: Three independent measurements showed consistent differences in running capacity. A total of 519 differential metabolites were identified, with 255 up-regulated and 264 down-regulated. The KEGG pathway analysis revealed a significant enrichment of the Purine Metabolism pathway (ITP-ATP) in the high running capacity group (*p* < 0.05). The ITP-treated group exhibited a significantly higher running capacity than the controls (*p* < 0.05), confirming the efficacy of dietary ITP supplementation. **Conclusions**: The running capacity of rats is influenced by the ITP-ATP pathway, and exogenous ITP administration through dietary intervention significantly improves running ability.

## 1. Introduction

Running capacity is a key indicator of an individual’s physical fitness and overall health status [1,2]. Physical activity not only enhances cardiopulmonary function, and improves muscle strength and endurance, but it also plays a vital role in regulating energy metabolism, alleviating oxidative stress, suppressing inflammatory responses, and promoting muscle adaptation [3,4,5]. However, natural differences in running capacities exist among individuals. Previous studies have analyzed the factors influencing these differences from various perspectives. For instance, from a genetic standpoint, DNA polymorphisms are significant contributors to variations in running capacity [6]. From a psychological perspective, mental health disorders have also been found to affect running performance [7]. Nevertheless, these factors cannot fully account for the variability in running capacity between individuals.

Metabolomics, as an essential branch of systems biology, utilizes high-throughput technologies to comprehensively analyze small-molecule metabolites within organisms, providing deep insights into metabolic networks and physiological states [8]. Running significantly alters the concentrations of various metabolites, which are small molecules generated through metabolic reactions in the body [9]. During physical activity, the concentrations of metabolites involved in energy metabolism and other metabolic pathways undergo continuous change [10]. With advancements in metabolomics technologies, researchers can more precisely detect the molecular responses triggered by running and comprehensively uncover running-induced changes in metabolite concentrations. These altered metabolites are recognized as critical signaling molecules that influence running capacity [11].

Metabolomics studies in mice have revealed that metabolic pathways such as linoleic acid, phenylalanine, and glycerophospholipid metabolisms significantly impact the running capacity of Alzheimer’s disease (AD) model mice, providing a theoretical basis for modulating metabolism in AD organisms through running [12]. In human studies, metabolomics findings have demonstrated that various metabolites related to energy metabolism (e.g., glycolysis and the tricarboxylic acid cycle) and redox metabolism (e.g., the pentose phosphate pathway) significantly influence running capacity [13].

Although numerous studies have explored the factors influencing differences in running capacity from various perspectives and using different methods, few have systematically analyzed these factors from a metabolomics perspective or validated their reliability. This study aims to employ untargeted metabolomics to analyze serum metabolite differences in rats with varying running capacities, to identify key metabolic pathways, and to validate the pathways’ effectiveness using key molecular markers. By doing so, this study seeks to uncover the underlying metabolic mechanisms influencing running capacity and provide new insights into the biological basis of running performance.

## 2. Methods

### 2.1. Materials and Equipment

The rat treadmill (model YLS-15A) was purchased from Jinan Yiyan Technology Development Co., Ltd. (Jinan, China); the Ultra-high Performance Liquid Chromatography (model ACQUITY UPLC I-Class), High-resolution Mass Spectrometry (model XevoG2-XS QTof), and Chromatographic column (model HSS T3 column) were purchased from Waters Company. The Centrifuge (model Legend Micro 17R, Shanghai, China) was purchased from Thermo Fisher Scientific; Water (LC-MS, Shanghai, China), Acetonitrile (LC-MS), and Formic acid (LC-MS) were also purchased from Thermo Fisher Scientific. Inosine Triphosphate (ITP) was purchased from Beijing Aoke Biotechnology Co., Ltd. (Beijing, China) [14].

### 2.2. Experimental Design

Twenty 8–10-week-old SPF-grade male Sprague-Dawley (SD) rats were purchased from Beijing Vitohe Laboratory Animal Technology Co., Ltd. (Beijing, China). Male Sprague-Dawley rats were selected for the initial metabolomics analysis to control for potential confounding effects of estrogen fluctuations in females, which may influence metabolic profiles during exercise. This approach aligns with previous studies investigating sex-independent metabolic responses to physical activity [15]. The experiments were conducted at the SPF-grade animal facility of Wannan Medical College. All procedures adhered to the relevant animal welfare and ethical guidelines outlined in the Regulations on the Management of Laboratory Animals at Wannan Medical College and were approved [WNMC-AWE-2024365] by the Institutional Animal Care and Use Committee (IACUC) of Wannan Medical College.

A total of 20 SDF rats were put through three repeated rat treadmill tests and screened into high and low running capacity groups. Four (4) rats from each running capacity group (High = 4, Low = 4) were then selected for the experiment. The selection criteria for running capacity were based on a rotary treadmill test. Each rat was given two exhaustion rest opportunities, with exhaustion defined as remaining stationary on the treadmill for more than 15 s, after which the rat received a one-minute rest period. The total running time and distance were recorded, and the rats with the highest cumulative running time and distance were classified into the high running capacity group, while those with the lowest were placed in the low running capacity group. The experiment was repeated three times to ensure result consistency and reliability.

Peripheral blood samples were then collected from the rats’ orbital regions to isolate serum, and metabolites were detected using liquid chromatography-tandem mass spectrometry (LC-MS/MS). After preprocessing the data, a principal component analysis (PCA) and a partial least squares discriminant analysis (PLS-DA) were employed to assess metabolic profile differences, and significant differential metabolites were identified [16,17]. Subsequently, a KEGG pathway enrichment analysis was performed on these metabolites to identify key metabolic pathways and validate their reliability.

### 2.3. Collection and Processing of Rat Serum Samples

Blood samples were collected one week after the completion of the treadmill exhaustion test. Blood was collected via orbital vein sampling. After centrifugation, serum was collected and quickly frozen for storage. A total of 8 samples were collected, divided into two groups, each with 4 replicates. Each serum sample (50 µL) was mixed with 150 µL of a methanol solvent, vortexed for 1 min at −20 °C, and left to stand overnight. The mixture was then centrifuged (2000 rpm, 4 °C, 20 min), and the supernatant was collected for a subsequent mass spectrometry analysis. Quality control samples were prepared during the sample processing to ensure the stability and accuracy of the analysis.

QC samples were generated by pooling 10 μL aliquots from each experimental serum sample. These pooled QCs were analyzed every 10 experimental injections throughout the LC-MS/MS run sequence. Metabolites with a coefficient of variation (CV) > 30% across the QCs were excluded to ensure data reliability. Instrument stability was further validated by calculating Pearson correlation coefficients (r) between the QC replicates, retaining only features with r > 0.90.

#### Liquid Chromatography-Mass Spectrometry Analysis Conditions (LC-MS/MS)

The Parameter Settings were as follows: Column Temperature: 40 °C; Sample Temperature: 10 °C; and Liquid Phase Flow Rate: 0.30 mL/min. The Mobile Phases were as follows: Phase A: Water + 0.1% formic acid (FA); and Phase B: Acetonitrile + 0.1% FA.

The Operating Conditions were as follows: Negative Ion Collection Mode: The IDA (Information-Dependent Acquisition) high-sensitivity scanning mode was used, with dynamic background subtraction enabled.

The Ion Source Parameters were as follows: Sheath Gas Flow Rate: 30; Gas1 Flow Rate: 50; Gas2 Flow Rate: 50; Temperature: 500 °C; Scanning Time: 15 min; and Scan Range: 60–1250 *m*/*z* for the primary scan, followed by 12 secondary scans for each primary scan. For Positive Ion Collection Mode, the same scanning mode and parameter settings were used [18].

**Figure 1:**
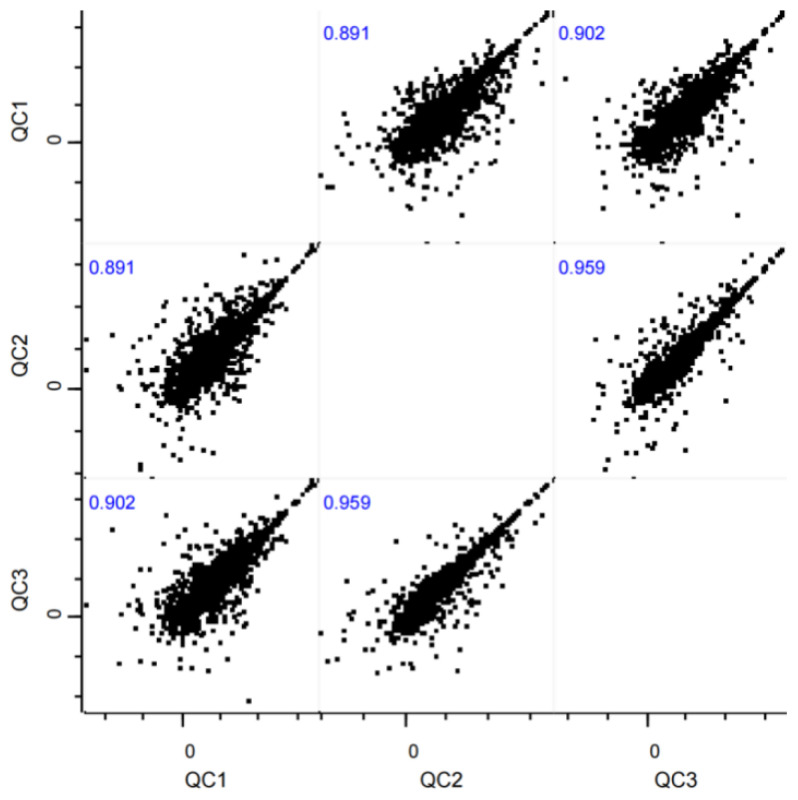
Correlation analysis of QC samples between high and low running capacity groups (n = 8 per group). Scatter plots depict log^2^-transformed metabolite intensity values from three independent experiments. Black points represent QC samples from the high and low running capacity groups. Pearson correlation coefficients (r > 0.80) indicate high intra-group consistency.

To evaluate the reproducibility of multiple biological experiments on the research samples, a correlation analysis was performed based on the intensity values of the commonly quantified metabolites across groups. The intensity values of the metabolites for each experimental group were log^2^-transformed and plotted on the x-axis and y-axis, respectively. The Pearson correlation coefficients for the commonly quantified metabolites between any two repeated experiments are shown in Figure 1. All correlation coefficients exceeded 0.80, indicating excellent consistency.

### 2.4. Metabolomics Data Extraction

Raw LC-MS/MS data were processed to extract metabolic features using Progenesis QI V2.3 software (Waters Corporation, Milford, MA, USA). Initially, raw spectra were imported and aligned to correct retention time shifts across the samples. Baseline noise reduction and peak detection were performed with a sensitivity threshold set to 10,000 counts, ensuring a robust identification of low-abundance metabolites. Features were filtered to retain those with coefficients of variation (CV) < 30% in the quality control (QC) samples, ensuring data reproducibility. Peak annotation was conducted by matching accurate mass-to-charge ratios (*m*/*z*) (±5 ppm) and retention times against the Human Metabolome Database (HMDB) and in-house spectral libraries. Isotopic peaks and adducts (e.g., [M + H]^+^, [M − H]^−^) were excluded using built-in algorithms. Data normalization was performed using total ion current (TIC) correction to minimize the batch effects. A three-dimensional matrix comprising metabolite intensities, retention times, and *m*/*z* values was generated for a downstream analysis. To ensure data quality, QC samples were injected at regular intervals (every 10 experimental samples) and evaluated using Pearson correlation coefficients (>0.80), confirming instrument stability. Missing values were imputed using half-minimum substitution for the metabolites detected in ≥80% of the samples. This rigorous workflow ensured the reliable extraction of 519 differential metabolites for the subsequent statistical and pathway analyses.

### 2.5. Metabolomics Data Analysis Methods

The serum data of rats were analyzed using Progenesis QI V2.3 software, generating a three-dimensional dataset in Excel that included peak-identified ions, peak matching, and peak alignment. Metabolites were identified using the HMDB (Human Metabolome Database, http://www.hmdb.ca, accessed on 20 October 2024) through the native QI (Waters) data processing software. The processed data were then imported into R for an orthogonal partial least squares discriminant analysis ((O)PLS-DA), which was used to visually represent the metabolic changes between the experimental groups.

Substances with a variable importance in projection (VIP) score >1 from the OPLS-DA model were selected to prioritize the metabolites contributing the most to group discrimination. Statistical significance was defined by a false discovery rate (FDR) < 0.05 after a Benjamini–Hochberg correction for multiple testing. Fold change (FC) thresholds (log^2^FC > 1.5 or <0.67) were applied to ensure biological relevance, consistent with prior metabolomics studies [19,20]. These criteria (VIP > 1, FDR < 0.05, and |log^2^FC| > 0.58) were established a priori to balance sensitivity and specificity. Candidates satisfying both a VIP >1 and an adjusted *p* < 0.05 were identified as potential biomarkers. Finally, a metabolic pathway enrichment analysis was performed using MetaboAnalyst 6.0.

### 2.6. ITP Intervention Experimental Method

Forty 8–10-week-old Sprague-Dawley (SD) rats, weighing 300–400 g, were selected for the experiment. The rats, evenly divided by sex, were randomly assigned to either the experimental group or the control group, with 20 rats in each group. The initial running capacity was evaluated using a rat treadmill exhaustion test to ensure no statistically significant difference between the two groups. The ITP group received an intragastric administration of 24.29 mg/kg.bw ITP (inosine triphosphate) daily for seven consecutive days. The control group received the solvent (saline) only. After the intervention, the running capacities of the rats were reassessed.

### 2.7. Data Analysis

Data were recorded and analyzed using SPSS 26.0 software. A statistical analysis was performed using the Mann–Whitney U test to determine significant differences between the groups.

## 3. Results

### 3.1. Rat Treadmill Test Analysis

The high running capacity group demonstrated significantly longer running times and distances compared to those of the low running capacity group in three repeated experiments, with statistically significant differences observed, as shown in Table 1.

### 3.2. Rat Serum Metabolomics Analysis

**Figure 2:**
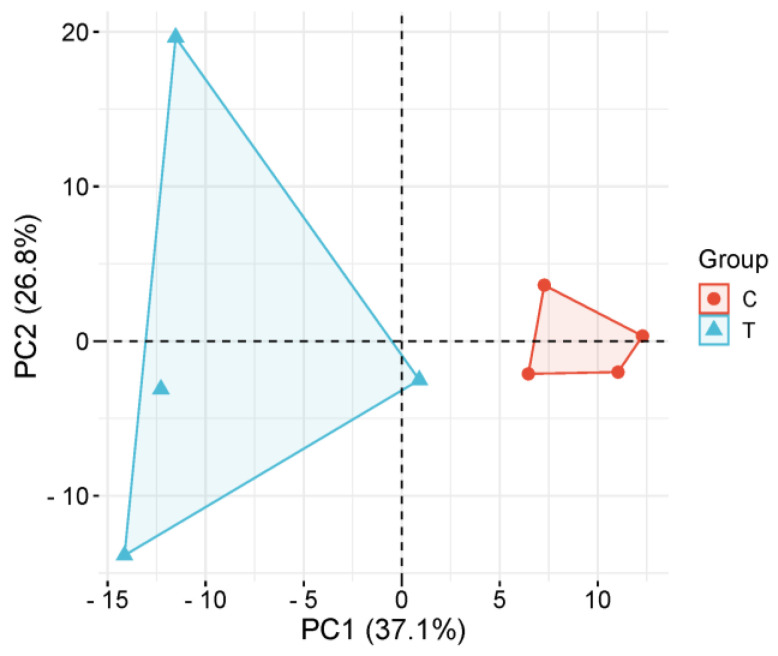
Principal component analysis (PCA) of serum metabolomes from high (red circles, n = 4) and low (blue triangles, n = 4) running capacity groups. PC1 (37.1%) and PC2 (26.8%) capture 63.9% of total variance. Group T (high capacity) exhibits broader dispersion compared to Group C (low capacity), reflecting metabolic heterogeneity post-intervention (permutational MANOVA: *p* < 0.001, F = 18.3). The 95% confidence ellipses confirm non-overlapping distributions.

The principal component analysis (PCA) revealed a clear separation between the high and low groups along the first two principal components (Figure 2). The model captured 63.9% of total metabolic variance (PC1: 37.1%, PC2: 26.8%), with PC1 primarily driving inter-group discrimination. The high running capacity specimens (red circles) clustered tightly in the positive PC1 quadrant (PC1 range: +5.2 to +9.8), demonstrating high intra-group consistency. In contrast, the low running capacity samples (blue triangles) exhibited broader dispersion across both the PC1 (−3.1 to +12.4) and PC2 (−8.6 to +15.2) dimensions, indicating enhanced metabolic heterogeneity following experimental intervention. The permutational MANOVA confirmed significant between-group dissimilarity (*p* < 0.001, F = 18.3), while the 95% confidence ellipses showed non-overlapping distributions, substantiating treatment-induced metabolic perturbation.

**Figure 3:**
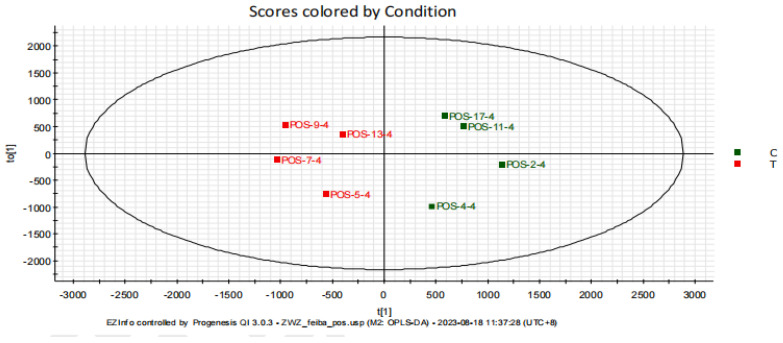
OPLS-DA score plot differentiating high (blue, n = 4) and low (red, n = 4) running capacity groups. Model parameters (R^2^Y = 89%, Q^2^ = 53%) demonstrate predictive validity. Permutation testing (200 iterations) with R^2^ intercept = 0.33 and Q^2^ intercept = −0.462 confirms minimal overfitting. Points represent individual rats, with separation along the predictive component (horizontal axis) driven by purine metabolism-related metabolites.

To eliminate the noise information unrelated to classification and to identify the reliable metabolites contributing to classification differences, an orthogonal partial least squares discriminant analysis (OPLS-DA) was applied to filter out the orthogonal signals unrelated to classification, generating the OPLS-DA model (Figure 3) [21]. The R^2^Y and Q^2^ values for the OPLS-DA model were 89% and 53%, respectively, indicating that the model has a certain level of predictive ability for the grouping.

**Figure 4:**
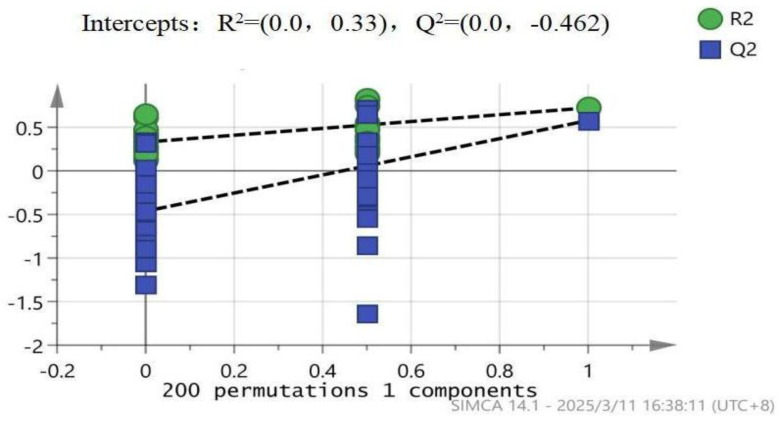
Validation of OPLS-DA model stability via permutation testing (200 permutations). Green circles (R^2^) and blue squares (Q^2^) represent permuted datasets. Dashed lines indicate original model values. R^2^ intercept = 0.33 and Q^2^ intercept = −0.462, suggesting robustness against overfitting. Data were derived from high (n = 4) and low (n = 4) running capacity groups.

To evaluate the reliability and overfitting risk of the OPLS-DA model, permutation testing revealed that the original OPLS-DA model (R^2^Y = 89%, Q^2^ = 53%) outperformed the permuted datasets (Figure 4), with the R^2^ intercept = 0.33 and the Q^2^ intercept = −0.462. Although negative Q^2^ values were observed in the permuted data, the R^2^ intercept remained below the empirical threshold of 0.4, indicating minimal overfitting. Cross-validation further supported the model’s robustness, with Q^2^ values > 0.5 across all folds. These results align with the established metabolomics standards [21], confirming the model’s reliability for a downstream analysis.

**Figure 5:**
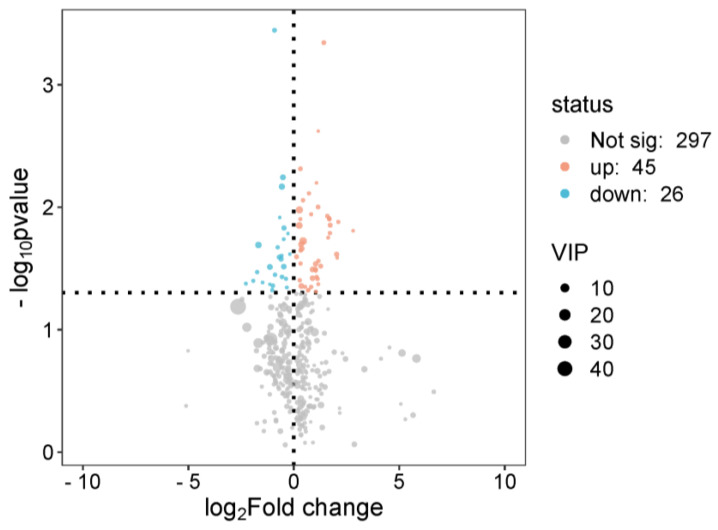
Volcano plot of serum metabolites between high (n = 4) and ow (n = 4) running capacity groups. Red points (VIP > 1, FDR < 0.05) indicate 519 significant metabolites (255 up-regulated, 264 down-regulated). Key metabolites (e.g., inosine triphosphate, tryptophan) are labeled. Dashed lines denote thresholds (log^2^ fold change > 1.5 or <0.67, FDR < 0.05).

Next, a metabolite volcano plot (Figure 5) was used to select metabolites with a VIP > 1 as differential metabolites. After a Benjamini–Hochberg correction for multiple testing, a total of 519 differential metabolites were identified using pre-specified thresholds (VIP > 1, FDR < 0.05, and |log^2^FC| > 0.58), with 255 up-regulated and 264 down-regulated. These criteria ensured robust discrimination between the high and low running capacity groups, while minimizing false positives. Ten differential metabolites from each group were listed (Table 2 and Table 3). Among these, the changes in metabolites such as tryptophan, Tocladesine, tryptophanol, inosine triphosphate, and uric acid have a certain impact on rat running capacity.

### 3.3. KEGG Pathway Analysis

**Figure 6:**
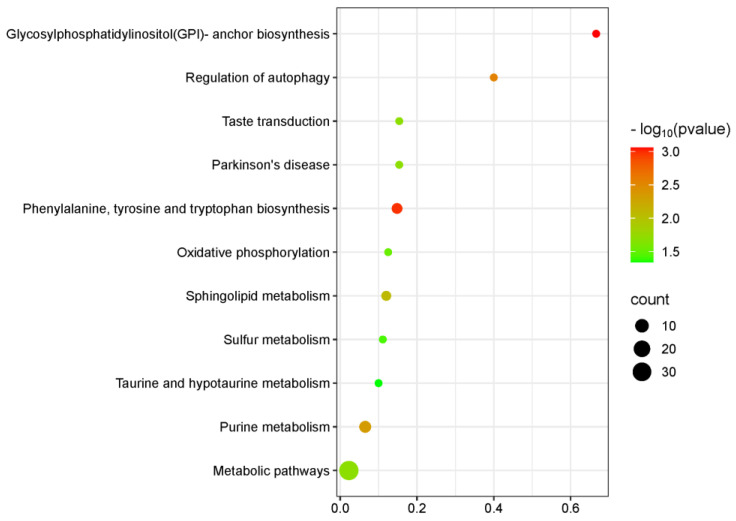
Bubble plot of KEGG pathway enrichment analysis for differential metabolites. Circle size reflects pathway impact, and color intensity indicates significance (−log^10^(*p*-value)). Glycerophospholipid metabolism (*p* = 0.003), purine metabolism (*p* = 0.007), and taurine metabolism (*p* = 0.012) are highlighted. Data were derived from high vs. low running capacity groups (n = 8).

**Figure 7:**
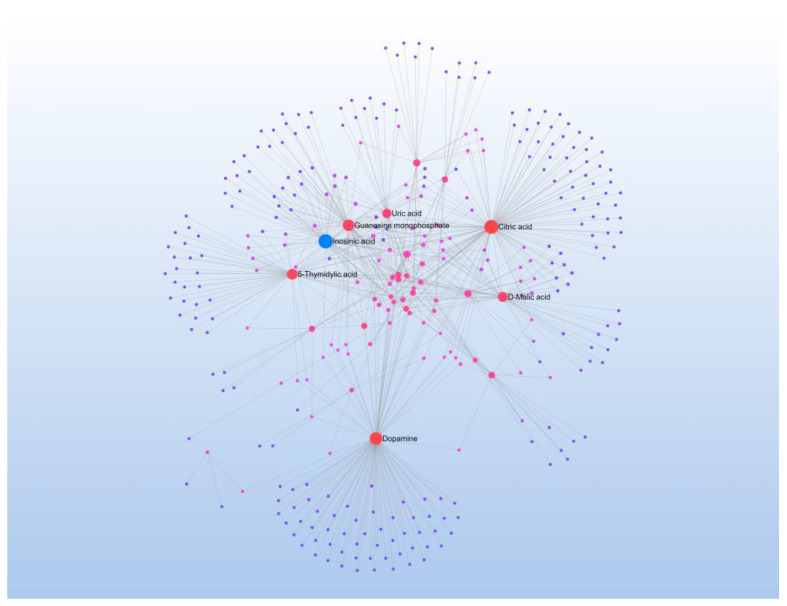
Cytoscape-generated interaction network of differential metabolites. Nodes represent metabolites (size: fold change; color: up-/down-regulation), and edges indicate biochemical interactions. Inosine triphosphate (ITP, red node) serves as a hub connecting purine metabolism (e.g., ATP, ADP) and energy pathways. Data were from high (n = 4) and low (n = 4) running capacity groups.

The differential metabolites were input into MetaboAnalyst 6.0 [22] for a pathway enrichment analysis, Among the metabolic pathways, significant changes were observed in glycerophospholipid, purine, and taurine and hypotaurine metabolism pathways (Figure 6). The enriched differential metabolites were then entered into the STRING database (http://string-db.org/ (accessed on 20 October 2024)) to obtain a metabolic interaction network, which was subsequently exported to Cytoscape software 3.10.1 for the construction of an interaction network (Figure 7). In the network, each node represents a metabolite, and the connecting lines indicate interactions between metabolites. The size of the nodes reflects the extent of expression differences, with larger nodes representing greater differences. The central metabolites with higher interaction connectivity, such as inosine triphosphate (ITP), are likely key points in the pathway. ITP was selected for a further in-depth analysis in this study.

**Figure 8:**
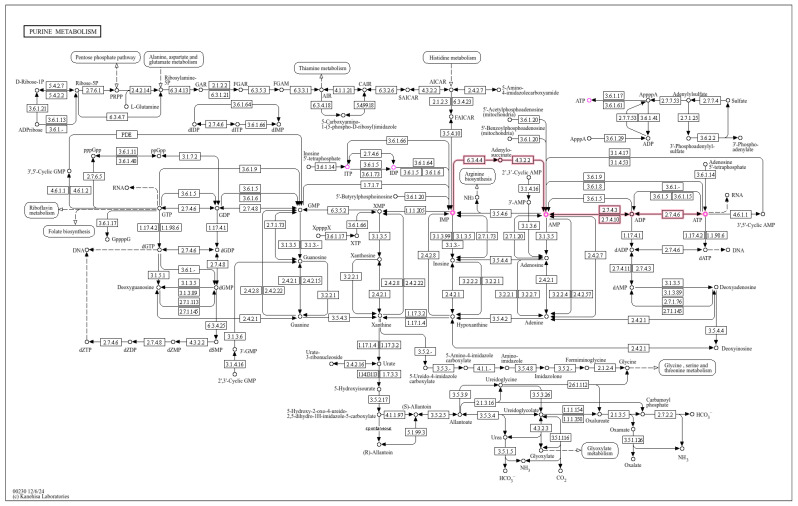
Schematic of the purine metabolism pathway (ITP-ATP axis) in high running capacity rats. Key intermediates (ITP, IDP, IMP, AMP, ADP, ATP) are labeled. Red arrows indicate up-regulation in the high group (n = 4) compared to the low group (n = 4). Pathway analysis highlights enhanced nucleotide turnover and ATP synthesis as drivers of improved exercise performance.

An interaction analysis was performed using MetaboAnalyst 6.0, focusing on the purine metabolism pathway. Figure 8 shows the key compounds in purine metabolism, including ITP-IDP-IMP-AMP-ADP-ATP (ITP-ATP) and their intermediate metabolites. These metabolites play critical roles in energy transfer, nucleic acid synthesis, signal transduction, and uric acid metabolism.

### 3.4. ITP Intervention Experiment

The results revealed, as shown in Table 4, that the difference in the running abilities of the rats before and after gavage was statistically significant (male *p* < 0.01, female *p* < 0.05).

## 4. Discussion

Running capacity is influenced by complex mechanisms involving multiple factors. Studies have shown significant differences in muscle metabolites and substrate metabolism during endurance running between genders. Women are naturally more suited to moderate-intensity running compared to men, primarily due to differences in substrate metabolism [15]. Additionally, research comparing professional athletes to individuals with lower physical fitness during running revealed that professional athletes exhibit greater metabolic flexibility, as evidenced by differences in blood lactate levels, fat oxidation, and carbohydrate oxidation [23]. These findings suggest that variations in metabolic pathways are critical factors influencing the running capacity of rats. In the present study, three repeated experiments (rat exhaustion tests) demonstrated that rats also exhibit natural differences in running capacity. This indicates that both human and animal running capacities inherently differ and are possibly affected by metabolic pathways.

### 4.1. Key Differential Metabolites and Their Functional Roles

Our metabolomics analysis identified 519 differential metabolites between the high and low running capacity groups, including 255 up-regulated and 264 down-regulated metabolites. Among these, inosine triphosphate (ITP) emerged as a central metabolite in the purine metabolism pathway. ITP, as a nucleotide triphosphate, is directly involved in ATP synthesis through the ITP-IDP-IMP-AMP-ADP-ATP conversion cascade. This pathway not only serves as a critical energy reservoir but also regulates nucleic acid synthesis and cellular signaling [24,25]. The upregulation of ITP in the high-capacity rats suggests enhanced nucleotide turnover and energy metabolism efficiency, which may directly contribute to prolonged running endurance.

Another notable up-regulated metabolite, tryptophan, plays a dual role in running physiology. Tryptophan is a precursor for serotonin synthesis, which modulates central fatigue perception [26], and its catabolism via the kynurenine pathway generates NAD+, a coenzyme essential for mitochondrial energy production [27]. Elevated tryptophan levels may thus mitigate fatigue and support sustained aerobic performance. Conversely, uric acid, a downstream product of purine metabolism, showed significant changes. While excessive uric acid accumulation is linked to oxidative stress [28], its moderate elevation in the high-capacity rats may reflect a balance between energy demand and antioxidant defense.

Among the down-regulated metabolites, phospholipids such as PE(20:1/22:6) and PC(16:0/22:6) exhibited reduced levels. These glycerophospholipids are key components of cellular membranes and signaling molecules. Their down-regulation may indicate membrane remodeling to adapt to running-induced oxidative stress or energy redistribution toward ATP synthesis [29]. Furthermore, the level of farnesyl triphosphate, involved in protein prenylation and cellular signaling, was significantly reduced. This could reflect a shift in metabolic priorities, favoring immediate energy production over long-term signaling investments during exhaustive running.

### 4.2. Metabolic Pathway Crosstalk and Network Dynamics

The interaction network analysis highlighted the centrality of the ITP-ATP pathway, with ITP acting as a hub metabolite connecting energy metabolism, redox regulation, and inflammatory responses. The enrichment of glycerophospholipid and taurine metabolisms further underscores the systemic nature of running adaptation. For instance, taurine, though not directly measured here, is known to stabilize membranes and enhance calcium handling in muscles, potentially synergizing with purine metabolism to improve contractile efficiency [30].

### 4.3. Validation Through ITP Intervention

The exogenous administration of ITP significantly enhanced running capacity in both the male and female rats, corroborating the metabolomics findings. This intervention likely amplified the endogenous ITP-ATP flux, thereby increasing ATP availability for muscle contraction and delaying fatigue. Notably, the sex-specific responses (a greater improvement in the males) align with prior studies on sex-dimorphic substrate utilization [15], suggesting a hormonal or enzymatic modulation of purine metabolism.

## 5. Limitations

Although this study reveals the impact of the ITP-ATP pathway on running capacity in rats, there are several limitations. First, the sample size in this study was limited to 20 rats, which may affect the statistical significance and generalizability of the results. Secondly, although three metabolic pathways were identified, only the purine metabolism pathway (ITP-ATP) was validated due to the lack of suitable drugs for glycerophospholipid metabolism and the taurine and hypotaurine metabolism pathways. Finally, this study was conducted solely in a rat model, and while it provides valuable biological insights, the applicability of these results to humans remains uncertain. Metabolic differences between species may influence the effectiveness of ITP intervention, and further studies are needed to validate these findings in humans.

## 6. Conclusions

Rats exhibit natural differences in running capacity. Rats with high running capacities show significantly more active purine metabolism pathways, with key metabolites such as ITP-IDP-IMP-AMP-ADP-ATP exhibiting significantly elevated levels. In this rat model, the exogenous supplementation of ITP can promote the activity of the purine metabolism pathway and enhance running capacity. However, due to species-specific metabolic differences, these findings should not be directly extrapolated to humans without further validation. Future studies should explore the translational potential of ITP intervention in preclinical models and clinical trials to assess its applicability in improving human exercise performance.

## Figures and Tables

**Table 1:** High and low running capacity groups across three repeated experiments [data are presented as mean ± SD for weight and median (Q_1_–Q_3_) for time and distance, n = 8].

Group	Weight (g)	First Time			Second Time			Third Time		
		Time (min)	Distance (m)	*p*	Time (min)	Distance (m)	*p*	Time (min)	Distance (m)	*p*
Low	441 ± 21	6.5 (3.9–10.8)	116.1 (67.4–197.2)	0.014 *	5.3 (2.2–8.2)	94.1 (33.8–146.5)	0.014 *	5.2 (4.2–6.8)	89.4 (71.6–121.9)	0.028 *
High	446 ± 23	30.3 (29.1–32.1)	564.6 (560.9–582.5)		28.9 (27.2–29.9)	537.9 (502.1–560.1)		30.8 (29.7–30.2)	557.6 (549.8–563.6)	

Note: * indicates *p* < 0.05 compared to the low running group.

**Table 2:** Metabolic differences in rats (up-regulated).

No.	Compound	Molecular Formula	Precise Molecular Weight (Da)	RT (min)	Score	*p* Value	Fold Change
1	1,2,10-Trihydroxydihydro-trans-linalyl oxide 7-O-beta-D-glucopyranoside	C_16_H_30_O_10_	382.18	12.09	34.1	<0.05	2.84
2	Tryptophan	C_11_H_12_N_2_O_2_	204.23	7.3	48.2	<0.05	2.12
3	Deacetylnomilinic acid	C_26_H_34_O_9_	490.22	6.91	37	<0.05	1.75
4	Inosine triphosphate	C_10_H_15_N_4_O_14_P_3_	574.06	9.90	36.8	<0.05	1.74
5	Edetic acid	C_10_H_16_N_2_O_8_	292.08	0.89	39.7	<0.05	1.65
6	Lactosylceramide (d18:1/26:1(17Z))	C_56_H_105_NO_13_	999.75	0.83	31.4	<0.05	1.62
7	Tetrahydrodeoxycortisol	C_21_H_34_O_4_	350.24	12.09	42.1	<0.05	1.59
8	Tocladesine	C_10_H_11_C_l_N_5_O_6_P	363.01	0.89	34.7	<0.05	1.56
9	Gamma-glutamyltyrosine	C_14_H_18_N_2_O_6_	310.11	4.34	37.6	<0.05	1.56
10	Chalepin acetate	C_21_H_24_O_5_	356.16	11.48	36.7	<0.05	1.51

**Table 3:** Metabolic differences in rats (down-regulated).

No.	Compound	Molecular Formula	Precise Molecular Weight (Da)	RT (min)	Score	*p* Value	Fold Change
1	PE(20:1(11Z)/22:6(4Z,7Z,10Z,13Z,16Z,19Z))	C_47_H_80_NO_8_P	817.56	11.35	37.6	<0.05	0.65
2	PC(16:0/22:6(4Z,7Z,10Z,13Z,16Z,19Z))	C_46_H_80_NO_8_P	805.56	13.53	35.1	<0.05	0.60
3	PC(o-16:0/20:4(8Z,11Z,14Z,17Z))	C_44_H_82_NO_7_P	767.57	11.37	34.1	<0.05	0.59
4	PC(18:0/22:6(4Z,7Z,10Z,13Z,16Z,19Z))	C_48_H_84_NO_8_P	833.59	13.54	36.3	<0.05	0.55
5	Farnesyl triphosphate	C_15_H_29_O_10_P_3_	562.09	4.12	35.6	<0.05	0.51
6	S-methyl-5-thio-D-ribulose 1-phosphate(2-)	HMDB0062647	785.57	13.52	33.2	<0.05	0.45
7	PC(22:4(7Z,10Z,13Z,16Z)/14:0)	C_44_H_80_NO_8_P	781.56	13.52	40.2	<0.05	0.41
8	PC(16:1(9Z)/22:6(4Z,7Z,10Z,13Z,16Z,19Z))	C_46_H_78_NO_8_P	803.54	11.51	53.1	<0.05	0.39
9	PC(18:0/18:3(6Z,9Z,12Z))	C_44_H_82_NO_8_P	783.57	12.46	38	<0.05	0.37
10	Aclarubicin	CSID397638	857.56	12.37	39.6	<0.05	0.36

**Table 4:** Comparison of running abilities in male and female rats before and after intervention [Data are presented as mean ± SD for weight and median (Q_1_–Q_3_) for time and distance, n = 40].

Gender	Group	Before Experiment				After Experiment			
		Weight (g)	Time (min)	Distance (m)	*p*	Weight (g)	Time (min)	Distance (m)	*p*
Female	Control group	328 ± 19	3.8 (2.6–7.5)	70.1 (43.7–142.2)	0.201	333 ± 22	3.6 (2.6–6.6)	64.5 (44.1–121.1)	0.039 *
	ITP group	329 ± 19	7.8 (3.1–9.3)	160.7 (53.1–176.1)		332 ± 16	10.9 (5.3–14.7)	206.9 (96.7–277.6)	
Male	Control group	359 ± 39	6.87 (2.2–9.1)	155.7 (38.4–190.3)	0.305	368 ± 34	5.7 (2.7–6.9)	103.9 (52.9–27.7)	0.009 **
	ITP group	353 ± 31	8.8 (3.1–11.9)	181.8 (58.8–268.7)		380 ± 38	15.9 (6.4–27.7)	299.9 (114.9–523.3)	

Note: * indicates a statistically significant difference compared to the control group (*p* < 0.05); ** indicates a highly statistically significant difference compared to the control group (*p* < 0.01).

## Data Availability

The data presented in this study are available on request from the corresponding author. (The data are not publicly available due to privacy).
